# Neuroprotective effect of cellular prion protein (PrPC) is related with activation of alpha7 nicotinic acetylcholine receptor (α7nAchR)-mediated autophagy flux

**DOI:** 10.18632/oncotarget.4953

**Published:** 2015-07-22

**Authors:** Jae-Kyo Jeong, Sang-Youel Park

**Affiliations:** ^1^ Biosafety Research Institute, College of Veterinary Medicine, Chonbuk National University, Jeonju, Korea; ^2^ Department of Bioactive Material Sciences and Research Center of Bioactive Materials, Chonbuk National University, Jeonju, Korea

**Keywords:** autophagy flux, alpha-7 nicotinic receptor, prpc, prion, Geotarget

## Abstract

Activation of the *alpha7* nicotinic acetylcholine receptor (α7nAchR) is regulated by prion protein (PrPC) expression and has a neuroprotective effect by modulating autophagic flux. In this study, we hypothesized that PrPC may regulate α7nAchR activation and that may prevent prion-related neurodegenerative diseases by regulating autophagic flux. PrP(106–126) treatment decreased α7nAchR expression and activation of autophagic flux. In addition, the α7nAchR activator PNU-282987 enhanced autophagic flux and protected neuron cells against PrP(106–126)-induced apoptosis. However, activation of autophagy and the protective effects of PNU-282987 were inhibited in PrPC knockout hippocampal neuron cells. In addition, PrPC knockout hippocampal neuron cells showed decreased α7nAchR expression levels. Adenoviral overexpression of PrPC in PrPC knockout hippocampal neuron cells resulted in activation of autophagic flux and inhibition of prion peptide-mediated cell death via α7nAchR activation. This is the first report demonstrating that activation of α7nAchR-mediated autophagic flux is regulated by PrPC, and that activation of α7nAchR regulated by PrPC expression may play a pivotal role in protection of neuron cells against prion peptide-induced neuron cell death by autophagy. These results suggest that α7nAchR-mediated autophagic flux may be involved in the pathogenesis of prion-related diseases and may be a therapeutic target for prion-related neurodegenerative diseases.

## INTRODUCTION

Alpha-7 nicotinic acetylcholine receptors (α7nAchR), also known as α7 nicotinic receptors, are a family of nicotinic acetylcholine receptors that regulate long-term memory and consist entirely of α7 subunits [1–3]. This receptor is distributed in the brain, endothelium, muscle and lymphocytes [2, 4–6]. α7nAchR are activated by post- and pre-synaptic excitation, mainly by increased calcium permeability [7, 8]. Recent work suggests that activation of these receptors is regulated by stress-inducible protein 1 through cellular prion protein (PrPC) signals [9]. In addition, activation of α7nAchR has a neuroprotective effect against misfolded protein-mediated neurodegenerative diseases, including Alzheimer's, and Parkinson's diseases [10–13]. Hung et al. found that autophagy-mediated amyloid beta (Aβ) clearance is regulated by activation of α7nAchR in SH-SY5Y neuroblastoma cells [13]. Another study showed that some α7nAchR agonists (AQW051 and PNU-282987) protects the brain from 1-methyl-4-phenyl-1,2,3,6-tetrahydropyridine (MPTP)-induced parkinsonism [10, 14]. In addition, α7 nAChR antagonist, methyllycaconitine blocked the nicotine-mediated neuroprotective effect in differentiated PC-12 cells [15]. These data suggest that activation of α7nAchR is regulated by PrPC expression and may have a neuroprotective effect against neurodegenerative disorders by interacting with PrPC signals.

PrPC is normal prion protein distributed in various tissues, including the lung, intestinal tract, and brain and is a glycosylated-phosphatidylinositol-anchored membrane protein involved with regulating neurosignals [16–20]. One study showed that PrPC may influence autophagic flux, although the molecular mechanisms are unclear [21]. Oh et al. suggested that PrPC protects hippocampal neuron cells against autophagic cell death caused by serum deprivation in *PrnP*^−/−^ hippocampal neuron cells [21]. Additionally, knockdown of PrPC expression in various cancer cells increases autophagy-mediated cell death [22]. However, one study suggested that PrPC has a neuroprotective effect associated with induction of autophagy against oxidative stress in hippocampal neuron cells [23]. Moreover, Shin et al. found that the increase of autophagic flux caused by depletion of PrPC is correlated with age in the hippocampus compared to that in normal mice [24]. The same study suggested that a PrPC deficiency may disrupt autophagic flux by blocking autophagosome-lysosomal fusion [24]. These results suggest that PrPC is a key factor in the regulation of autophagic flux in the brain, although the relationship between PrPC and autophagic flux is unclear.

Autophagy is well known process for degrading cytoplasmic components via the lysosomal pathway [25–28]. During autophagy, LC3-I (cytosolic form) is conjugated to PE (phosphatidylethanolamine) and form LC3-II (LC3-PE conjugated form) [29, 30]. It is composited to autophagosomal membranes, and which is degraded by lysosomal digestion after the fusion of Avs (autophagic vacuoles) with lysosomes [31, 32]. Thus, increase of LC3-II indicated that activating autophagy. But some paper showed that inhibition of autophagy also increased LC3-II form [33]. A block of lysosomal fusion with AVs inhibited degradation of LC3-II forms [33]. Also, autophagic flux is inhibited, the level of p62, an ubiquitin- and LC3-binding protein, is accumulated [34, 35]. Thus, the comparative analysis about the LC3-II/LC3-I ratio and p62 accumulation for the detection of autophagic flux is necessary. And, some paper showed that accumulation of p62 is detected in neurodegenerative disorders including Parkinson disease, Alzheimer disease and Huntingtin aggregates in Huntington disease [36–38]. In addition, some studies have suggested that activating this process plays a pivotal role in various conditions, including adaptation to starvation and anti-cancer and neuroprotective effects [39–44]. It is well known that starvation stimulates the sirtuin family of deacetylases, thereby activating autophagic flux [45–47]. In addition, activation of autophagic flux increases Aβ clearance and protects neuronal cells against Aβ-mediated neurotoxicity by activating α7nAchR in SH-SY5Y neuroblastoma cells [13]. However, a defect in α7nAchR signaling may impair autophagic flux, thereby suppressing clearance of Aβ, leading to increased neurotoxicity [13]. These results suggest that the regulation of autophagic flux is a key mechanism for preventing neural dysfunction. In particular, activating autophagic flux through α7nAchR signaling may prevent misfolded protein-mediated neurodegenerative disorders including Alzheimer's, Parkinson's, and prion diseases. Our previous study showed that overexpressing PrPC [48] and activating autophagy protects neuron cells against PrP(106-126)-mediated neurotoxicity [43, 44], respectively. However, the relationship between PrPC and autophagy and prion peptide-mediated neurotoxicity has not been reported.

Thus, the present study focused on the relationship between PrPC expression and the regulation of autophagic flux during PrP(106-126)-mediated neurotoxicity and estimated the influence of α7nAchR signaling. The results show that activating α7nAchR upregulates autophagic flux and protects hippocampal neuron cells against PrP(106-126)-mediated neurotoxicity. However, the protective effect and upregulation of autophagic flux caused by activating α7nAchR was suppressed by decreasing PrPC gene expression. These results suggest that α7nAchR-mediated autophagic flux may be regulated via PrPC expression and that regulating PrPC expression is applicable as a therapeutic strategy for neurodegenerative disorders including prion disease.

## RESULTS

### Regulation of α7nAchR activity influences PrP(106-126)-mediated neurotoxicity by upregulating autophagic flux in primary neuron cells

**Figure 1:**
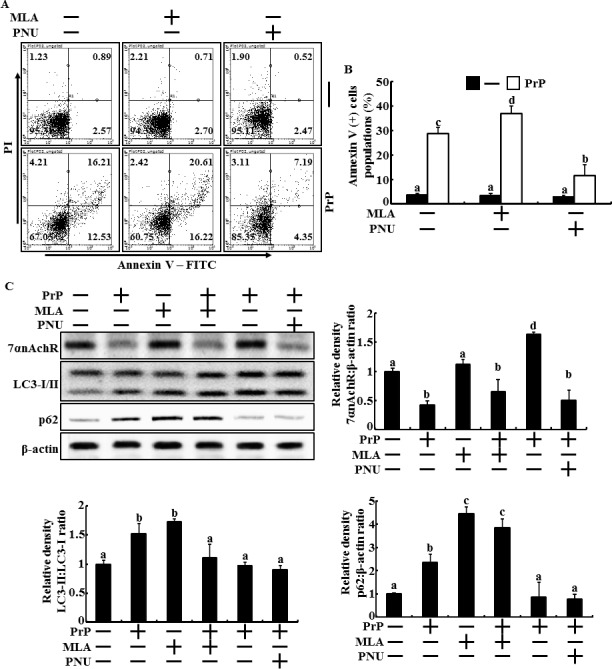
Regulation of α7nAchR activity influenced to autophagic flux and prevents primary neuron cells from PrP(106-126) treatment **A.** Primary neuron cells were treated with PNU-282987 (1 μM, 12 hr) or MLA (50nM, 12hr) and then exposed to 50 μM of PrP (106-126) for 24 h. Cell viability was measured by the Annexin V assay. **B.**, Bar graph indicated that the averages of annexin V positive cells. **C.**, Western blot assay in primary neuron cells treated as described in A. The treated cells were assessed for LC3-1/II, p62 and α7nAchR production by Western blot analysis. Results were normalized with β-actin. Expression levels were determined by western blot band quantifications and densitometric values are shown beside the blot.

Our previous study suggested that activating autophagy protects neuronal cells against PrP(106-126)-mediated neurotoxicity [43, 44]. Another study showed that activating α7nAchR upregulates autophagic flux and protects SH-SY5Y cells against Aβ-induced neurotoxicity [13]. However, the influence of α7nAChR in prion-mediated neurotoxicity has not been reported. Thus, we determined whether activating α7nAChR upregulates autophagic signaling during PrP (106-126)-induced apoptosis (Figure 1). Primary cultured neuronal cells were exposed to the α7nAChR inhibitor MLA or the α7nAChR activator PNU-282987 for 12 hr and were treated with 50 μM PrP (106-126) for 24 hr. The Annexin V assay results show that the number of apoptotic cells decreased after PNU-282987 treatment in the PrP(106-126)-treated groups (Figure 1A and 1B). Additionally, the number of Annexin V-positive cells increased following MLA treatment in PrP (106-126)-treated cells (Figure 1A and 1B). These data show that activating α7nAChR signaling protects neuron cells against prion-mediated neurotoxicity.

One study suggested that activating α7nAChR may upregulate autophagic flux; thus, we determined whether activating α7nAChR influenced autophagic flux in PrP(106-126)-treated cells (Figure 1C). Cells were pre-treated with MLA (50 nM, 12 hr) or PNU-282987 (1 μM, 12 hr) and were then exposed to 50 μM PrP (106-126) for 12 h. that the results show that protein levels of α7nAChR decreased and those of LC3-II/LC3-I ratio and p62 increased (autophagic flux inhibition marker) following PrP (106-126) treatment. Additionally, MLA reinforced the inhibition of autophagic signals caused by PrP(106-126) treatment. However, PNU-282987 increased LC3-II/LC3-I ratio and decreased p62 protein levels, indication of activating autopagic flux, in the PrP(106-126)-treated group, but α7nAChR expression did not change (Figure 1C). These results suggest that activating α7nAChR signaling protects neuron cells against PrP(106-126)-mediated apoptosis by upregulating autophagic flux.

**Figure 2:**
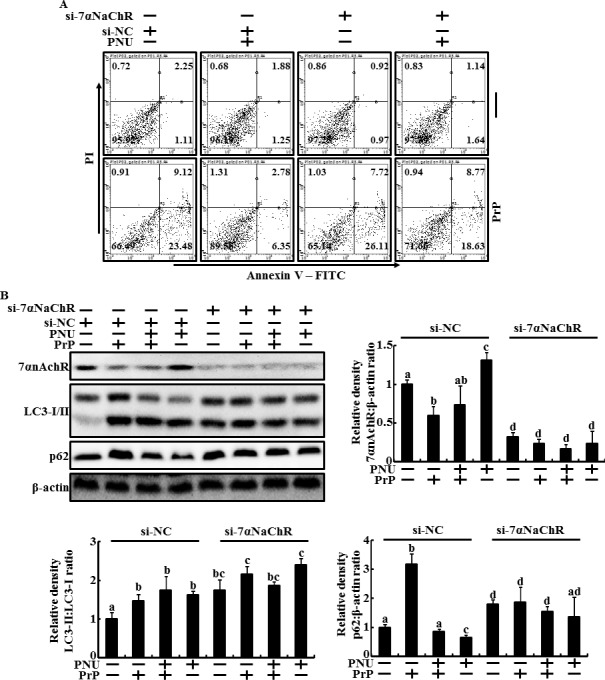
Knockdown of α7nAchR inhibited the PNU-282987-mediated neuroprotective effect and autophagic flux in primary neuron cells **A.**, α7nAchR siRNA (si-α7nAChR) or Negative control siRNA (NC) transfected primary neuron cellsls were treated with PNU-282987 (1 μM, 12 hr) and then exposed to 50 μM of PrP (106-126) for 24 h. Cell viability was measured by the Annexin V assay. **B.**, Western blot assay in primary neuron cells treated as described in A. The treated cells were assessed for LC3-I/II, p62, PrPC and α7nAchR production by Western blot analysis. Results were normalized with β-actin. Expression levels were determined by western blot band quantifications and densitometric values are shown beside the blot. The bar graph indicates the mean ± S.E.M. (*n* = 3).

To verify that α7nAChR plays a protective role in PrP(106-126)-treated neuron cells via activation of autophagic flux, we suppressed α7nAChR gene expression using an α7nAChR RNAi oligomer in primary neuron cells treated with PrP(106-126) and PNU-282987 as a α7nAChR activator. The number of Annexin V-positive cells no changed in α7nAChR RNAi oligomer (α7nAChR siRNA, si-α7nAChR) transfected cells. However, treatment of PrP(106-126) increased apoptotic cells populations in si-α7nAChR transfected cells compared to negative control RNAi oligomer (si-NC) transfected cells (Figure 2A). In addition, the protective effect of PNU-282987 against PrP(106-126)-mediated neurotoxicity inhibited by knockdown of α7nAChR in primary neuronal cells (Figure 2A). Additionally, Western blot analysis showed that the treatment of si-α7nAChR inhibited α7nAChR protein levels and increased LC3-II/LC3-I ratio and p62 protein levels compared to those in negative control RNAi oligomer (si-NC)-treated cells (Figure 2B). Also, treatment of si-α7nAChR increased the inhibition of autophagic flux in PrP(106-126)-treated cells (Figure 2B). Collectively, these data suggest that regulation of α7nAChR signaling influenced to prion-mediated neurotoxicity via modulation of autohpagic flux pathway.

### Depleting PrPC and PrP(106-126) inhibits α7nAChR expression and autophagic flux in hippocampal neuron cells

**Figure 3:**
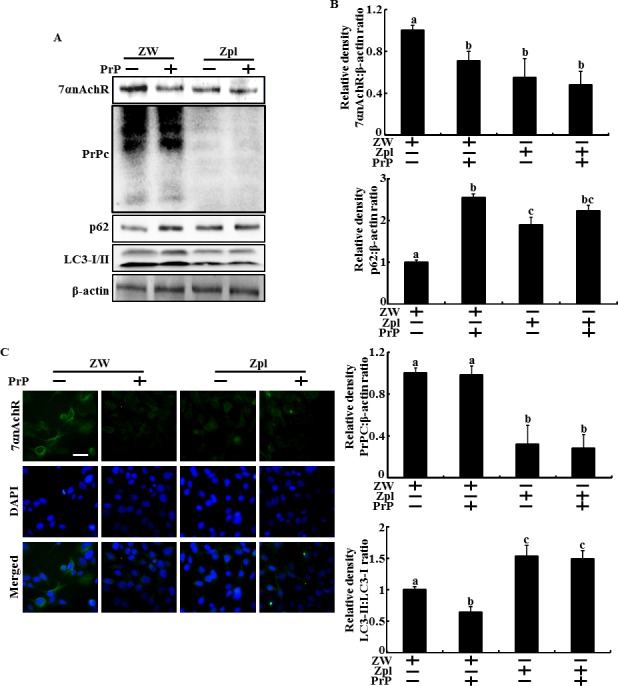
Depletion of PrPC and PrP(106-126) inhibited the alpha 7 nAchR expression and autophagic flux in hippocampal neuron cells **A.** and **B.**, ZW 13-2 and Zpl 3-4 cells were treated with prion peptide (50μM, 12h). The treated cells were assessed for LC3-I/II, p62, PrPC and α7nAchR production by Western blot analysis. Results were normalized with β-actin. Expression levels were determined by western blot band quantifications and densitometric values are shown beside the blot. The bar graph indicates the mean ± S.E.M. (*n* = 3). **C.**, Representative images of Immunocytochemistry in ZW 13-2 and Zpl 3-4 cells treated as described in **A.** The treated cells were immunostained with DAPI (blue) and α7nAchR antibody (green) and fluorescence was examined. Magnification 400×, scale bar = 20 μm.

One study showed that depleting PrPC blocks activation of α7nAChR signaling and inhibits autophagic flux signals. However, the relationship between autophagy and PrPC/α7nAChR signals in prion-mediated neurotoxicity is unknown. Thus, we investigated whether PrPC expression influences α7nAChR-mediated autophagic flux signaling in PrP(106-126)-treated neuronal cells. We cultured the mouse hippocampal neuron cell lines ZW 13-2 and Zpl 3-4, which were established from the hippocampus of ICR (*Prnp*^+/+^) and Zürich I *Prnp*^−/−^ mice, respectively. Western blot assays showed that PrP(106-126) treatment decreased LC3-II/LC3-I ratio and α7nAChR protein levels increased p62 protein levels in ZW 13-2 cells. In addition, P62 protein levels increased in PrPC-knockout Zpl 3-4 cells compared to those in ZW 13-2 cells. Zpl 3-4 cells decreased the α7nAChR protein levels compared to ZW-13-2 cells (Figure 3A and 3B). Consistent with these results, fluorescence microscopy showed that PrP(106-126) treatment or depleting PrPC expression resulted in low fluorescence (Figure 3C). These data indicate that PrPC expression plays a main role in α7nAChR-mediated autophagic flux in PrP(106-126)-treated hippocampal neuron cells.

### PrPC depletion influences α7nAChR-mediated autophagic flux signaling in PrP(106-126)-treated hippocampal neuron cells

**Figure 4:**
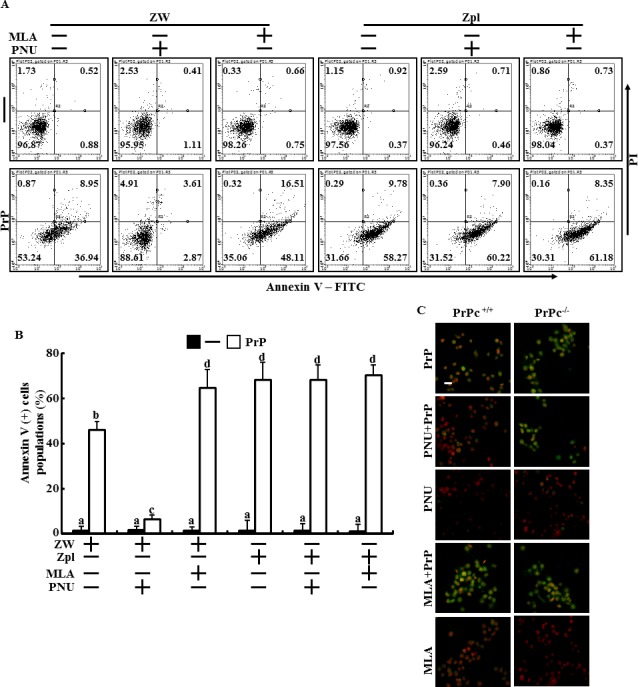
Regulation of alpha 7 nAchR activity influenced to the protective effect of PrPC against PrP(106-126)-mediated neurotoxicity **A.**, ZW 13-2 and Zpl 3-4 cells were treated with PNU-282987 (1 μM, 12 hr) or MLA (50nM, 12hr) and then exposed to 50 μM of PrP (106-126) for 24 h. Cell viability was measured by the Annexin V assay. **B.**, Bar graph indicated that the averages of annexin V positive cells. **C.**, Representative immunofluorescence images of TUNEL-positive (green) ZW 13-2 and Zpl 3-4 cells pre-treated with PNU-282987 (1 μM, 12 hr) or MLA (50nM, 12hr) and then exposed to PrP(106-126). The cells were counterstained with propidium iodide (red) to show all cell nuclei. Magnification 400×, scale bar = 20 μm.

**Figure 5:**
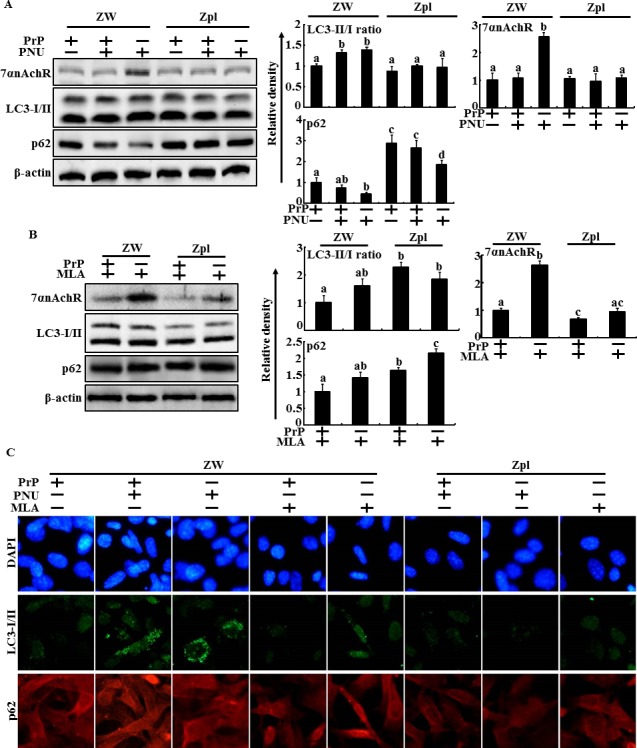
PrPC depletion influence to alpha 7 nAchR-mediated autophagic flux in PrP(106-126)-treated hippocampal neuron cells **A.** and **B.**, ZW 13-2 and Zpl 3-4 cells were treated with PNU-282987 (1 μM, 12 hr) or MLA (50nM, 12hr) and then exposed to Prion peptide (50μM, 12h). The treated cells were assessed for LC3-I/II, p62, PrPC and α7nAchR production by Western blot analysis. Results were normalized with β-actin. Expression levels were determined by western blot band quantifications and densitometric values are shown beside the blot. **C.**, Cells were treated with PNU-282987 (1 μM, 12 hr) or MLA (50nM, 12hr) and then exposed to Prion peptide (50μM, 12h). The treated cells were immunostained with DAPI (blue) and α7nAchR antibody (green) and fluorescence was examined. Magnification 400×, scale bar = 20 μm.

To verify that α7nAChR plays a protective role in PrP(106-126)-treated neuron cells via PrPC expression and to determine whether the neuroprotective effect of α7nAChR/PrPC signaling is related with autophagic flux, cell viability and the signaling pathways were evaluated in ZW 13-2 and Zpl 3-4 cells treated with PrP(106-126) and either PNU-282987 as a α7nAChR activator or MLA as a α7nAChR suppressor. The number of Annexin V-positive cells increased in Zpl 3-4 cells compared to that in ZW 13-2 cells after PrP(106-126) treatment (Figure 4A and 4B). Additionally, PNU-282987 protected ZW 13-2 cells against PrP(106-126)-induced apoptosis but the protective effect of PNU-282987 was inhibited in Zpl 3-4 cells. MLA increased PrP(106-126)-mediated neurotoxicity in ZW 13-2 cells, whereas it's effect was blocked in Zpl 3-4 cells (Figure 4A and 4B). These results were further confirmed by measuring TUNEL fluorescence (Figure 4C). A Western blot analysis showed that the PNU-282987 and PrP(106-126) co-treated group had increased LC3-II/LC3-I ratio and decreased p62 protein levels compared to those in the PrP(106-126) only treated group of ZW 13-2 cells (Figure 5A). However, these changes were not observed in Zpl 3-4 cells. The MLA and PrP(106-126) co-treated group showed increased p62 protein levels compared to those in the PrP(106-126) treated group (Figure 5B). Consistent with these findings, immunocytochemistry showed that PNU-282987 and PrP(106-126) co-treated cells had increased LC-3 protein levels (green fluorescence) but decreased p62 protein levels (red fluorescence) compared to those in the PrP(106-126) treatment group of ZW 13-2 cells; however, these changes were blocked in Zpl 3-4 cells (Figure 5C). These results suggest that α7nAChR signaling has a neuroprotective effect against PrP(106-126)-mediated neurotoxicity by upregulating autophagy and that these protective effect are influenced by PrPC expression in neuron cells.

**Figure 6:**
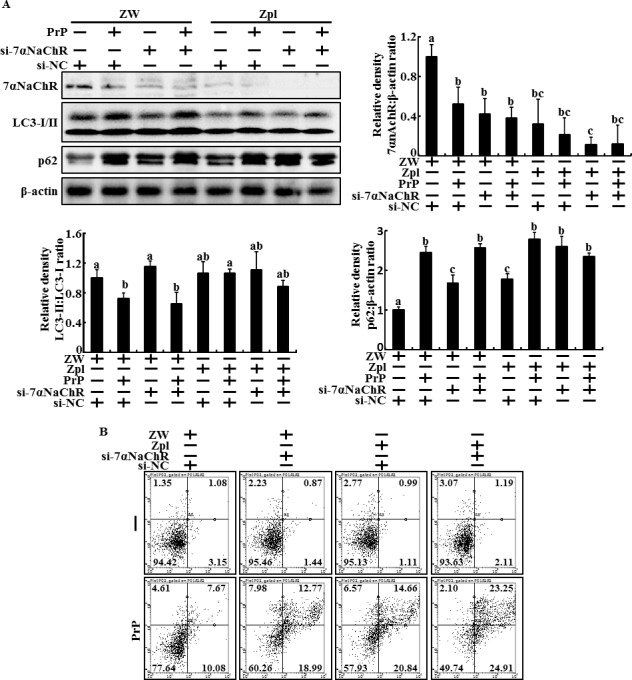
α7nAchR-mediated autophagic flux regulates by PrPC expression in PrP(106-126)-treated Hippocampal neuron cells **A.**, α7nAchR siRNA (si-α7nAChR) or Negative control siRNA (NC) transfected ZW 13-2 and Zpl 3-4 cells were incubated with 50 μM PrP. The treated cells were assessed for LC3-I/II, p62, PrPC and α7nAchR production by Western blot analysis. Results were normalized with β-actin. Expression levels were determined by western blot band quantifications and densitometric values are shown beside the blot. The bar graph indicates the mean ± S.E.M. (*n* = 3). **B.**, Annexin V assay in ZW 13-2 and Zpl 3-4 cells treated as described in A.

To verify this hypothesis, we knockdown α7nAChR gene expression using an α7nAChR RNAi oligomer to determine whether PrP(106-126)-induced neurotoxicity was related with PrPC expression or α7nAChR-mediated autophagy. A Western blot analysis showed that the α7nAChR RNAi oligomer (α7nAChR siRNA) treatment inhibited α7nAChR protein levels and increased LC3-II and p62 protein levels compared to those in negative control RNAi oligomer-treated ZW 13-2 cells (Figure 6A). However, these changes were not observed in Zpl 3-4 cells (Figure 6A). Additionally, PrP(106-126)-treated cells (ZW 13-2 and Zpl 3-4 cells) showed inhibited autophagic flux signals (Figure 6A). Knockdown of α7nAChR increased the inhibition of autophagic flux in PrP(106-126)-treated ZW 13-2 cells (Figure 6A). However, α7nAChR siRNA did not influence the PrP(106-126) treatment in Zpl 3-4 cells (Figure 6A). An increase in the number of PrP(106-126)-induced Annexin V-positive cells was observed in α7nAChR siRNA transfected ZW 13-2 cells, whereas this change was not observed in PrP(106-126)-treated Zpl 3-4 cells (Figure 6B). Collectively, these data suggest that PrPC activates autophagic flux by regulating α7nAChR signaling and partially protects neuron cells against prion-induced neurotoxicity.

### Upregulation of PrPC protects Zpl 3-4 cells against PrP(106-126)-induced apoptosis by activating α7nAChR-mediated autophagic signaling

**Figure 7:**
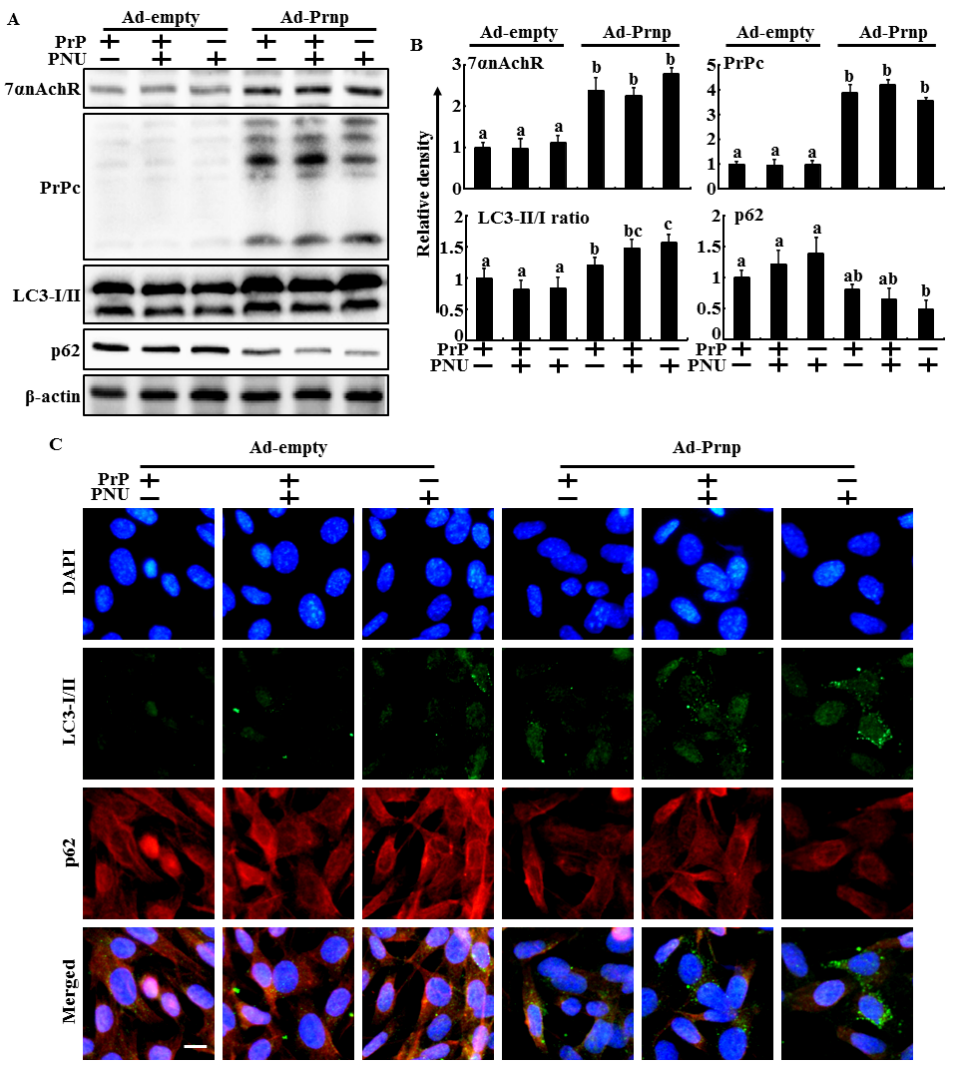
Overexpression of PrPC restored the autophagc effect caused by alpha 7 nAchR in PrPC-deficient neuron cells **A.**, **B.**, AD-*Prnp* or AD-empty transfected Zpl 3-4 cells were incubated with 50 μM PrP (106-126) for 12h after exposure α7nAchR agonist (PNU-282987, 1μM, 12h) treatment. The treated cells were assessed for α7nAchR, PrPC, LC3 and p62 production by Western blot analysis. Results were normalized with β-actin. Expression levels were determined by western blot band quantifications and densitometric values are shown beside the blot. The bar graph indicates the mean ± S.E.M. (*n* = 3). **C.**, Representative images of Immunocytochemistry in Zpl 3-4 cells treated as described in A-B. Scale bar denotes 20 μm.

**Figure 8:**
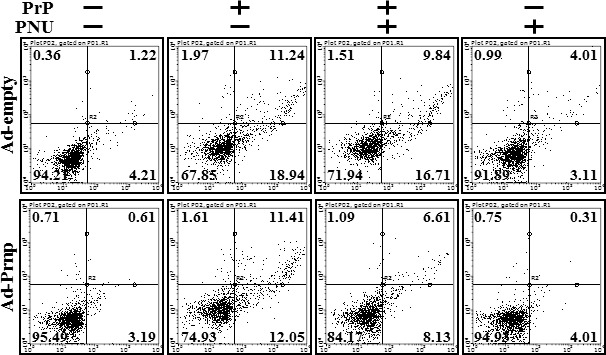
Overexpression of PrPC rescue the protective effect of alpha 7 nAchR in PrPC-deficient neuron cells AD-*Prnp* or AD-empty transfected Zpl 3-4 cells were incubated with 50 μM PrP (106-126) for 12h after exposure α7nAchR agonist (PNU-282987, 1μM, 12h) treatment. Cell viability was measured by the Annexin V assay. PNU; PNU-282987.

To verify that PrPC plays a protective role in neuron cells exposed to prion peptide by activating α7nAChR-mediated autophagic flux, the recombinant adenovirus-expressing full length *Prnp* gene (Ad-*Prnp*) was utilized to overexpress the *Prnp* gene in PrP(106-126)-treated Zpl 3-4 cells. Transfection of Zpl 3-4 cells with Ad-*Prnp* resulted in PrPC overexpression compared to that in Ad-empty transfected cells (Figure 7A and 7B). Ad-*Prmp* and Ad-empty transfected cells were pre-treated with PNU-282987 (1 μM, 12 hr) and then exposed to 50 μM PrP (106-126) for 12 hr. The result showed that overexpression of PrPC increased α7nAChR protein expression level and decreased p62 protein level in PrP(106-126)-treated cells. In addition, Ad-*Prnp* transfected cells had activated autophagic flux signals in response to PNU-282987, whereas Ad-empty transfected cells showed no change in LC3-II/LC3-I ratio or p62 expression level after PrP(106-126) treatment. Consistent with these results, immunocytochemistry showed that PNU-282987 restored autophagic flux in Ad-*Prnp* transfected cells (Figure 7C). The Annexin V assay showed that transfection of Ad-*Prnp* at a multiplicity of infection (MOI) of 500 inhibited PrP (106-126)-induced apoptosis compared to that in cells transfected with Ad-empty at a MOI of 500. PNU-282987 enhanced the protective effect of PrPC expression on PrP (106-126)-mediated neuronal cell death (Figure 8). These data indicate that overexpression of PrPC plays a protective role against prion peptide-induced neuron cell death by upregulating α7nAChR-mediated autophagy signaling.

## DISCUSSION

Our results demonstrate that activating α7nAChR prevented prion-mediated neuronal damage by activating autophagic flux and that inducing α7nAChR-mediated autophagic flux regulates PrPC expression in neuron cells. A Notably, activation of autophagic flux caused by α7nAChR was related to PrPC expression in neuron cells, which, in turn, conferred neuroprotection.

Some studies have reported that activating α7nAChR regulates cholinergic signaling and may lead to recover cognitive function in Alzheimer's disease models [13, 49, 50]. Nicotine and A-582941, which are α7nAChR agonists, protect neurons from Aβ-induced neuronal damage by upregulating the α7nAChR signaling pathway [50, 51]. ABT-107, which is a α7nAChR agonist, also prevents neurotoxicity induced by l-dopa-induced dyskinesia [52]. Neuronal cholinergic receptors are reduced in patients with Alzheimer's disease; particularly, α7nAChR expression decreases 32% [53]. Consistent with this finding, we showed here that PrP(106-126)-treated cells had decreased viability (Figure 1A and 1B) and α7nAChR protein expression in primary neuron cells (Figure 1C). In addition, the α7nAChR agonist PNU-282987 protected neuron cells from PrP(106-126), whereas treatment with the α7nAChR antagonist MLA enhanced PrP(106-126)-mediated neurotoxicity (Figure 1A and 1B), although the α7nAChR protein expression did not change (Figure 1C). These data support the hypothesis that regulation of α7nAChR may influence prion-mediated neurotoxicity in neuron cells.

One study reported that α7nAChR activity is regulated by PrPC expression [9], whereas other studies suggest that PrPC has a protective effect against neuronal damage [48], including prion peptide-mediated neurotoxicity. Also, neuroprotection is associated with autophagic flux signals [43, 44] and the protective effect were inhibited by PrPC depletion in hippocampal cells [23]. One paper showed that PrPC-depleted cells show increased expression of LC3-II, autophagy marker protein levels, and autophagosomes under serum starved conditions [21]. This accumulation of LC3-II is inhibited by transfecting the PrPC gene into PrPC knockout neuron cells [21]. These results indicate that PrPC may downregulate autophagic flux in neuron cells. However, Oh et al. suggested that PrPC knockout neuron cells have impaired autophagic flux caused by oxidative stress, whereas wild-type neuron cells exposed to oxidative stress increase autophagic flux [23]. The main mechanism of misfolded protein-mediated neurotoxicity is oxidative stress [54, 55]. Prion-mediated neurotoxicity is also mainly related with oxidative stress [56–58]. Our results show that the PrPC knockout Zpl 3-4 hippocampal neuron cells increased PrP(106-126)-mediated neurotoxicity compared to that of ZW 13-2 cells. In addition, Zpl 3-4 cells had increased p62 protein levels and LC3-II/LC3-I ratio compared to those of ZW 13-2 cells, but PrP(106-126) has no effect in Zpl 3-4 cells (Figure 3A and 3B). Thus, we hypothesize that PrP(106-126) treatment or depleting the PrPC gene inhibits autophagic flux and that PrPC may prevent prion peptide-mediated neurotoxicity by upregulating autophagic flux in neuron cells.

Autophagy is the main lysosomal catabolic pathway for recycling and degrading old cell organelles [25, 59]. The main function of autophagy is facilitated adaption of cells exposed to microenvironments, including caloric restriction, tumor metastasis, immune reactions, ischemic heart disease, and neurodegeneration [60–62]. One study suggested that activating autophagy may play a neuroprotective role in Alzheimer's disease and is related with α7nAChR signaling [13]. That same study showed that inhibiting α7nAChR signaling or depleting ATG7 gene enhanced Aβ-induced neurotoxicity [13]. Consistent with this finding, our results show that PNU-282987, an α7nAChR agonist, increased LC3-II/LC3-I ratio and decreased p62, a marker of activated autophagic flux, and that α7nAChR antagonist or α7nAChR RNA oligomer treatment inhibited autophagic flux in primary cultured neuron cells (Figures 1C and 2C). In addition, PNU-282987 protected neuron cells against PrP(106-12)-mediated neurotoxicity and MLA enhanced PrP(106-126)-mediated neurotoxicity (Figure 1A, 1B and 2A). These results also similarly showed in ZW 13-2 cells (Figures 4). However, the α7nAChR-mediated autophagic flux and protective effect disappeared in PrPC knockout Zpl 3-4 cells (Figures 5 and 6). In addition, we found that depleting PrPC decreased α7nAChR expression (Figure 3A and 3B). Thus, we hypothesize that PrPC is a key factor in the regulation of the α7nAChR pathway and is related with autophagic flux and neuroprotection against prion-mediated neurotoxicity.

PrPC expression demonstrated that PNU-282987 did not inhibit prion peptide-mediated neuronal apoptosis and blocked autophagic signals, indicating that activating the α7nAChR pathway has a neuroprotective effect related with PrPC expression. In addition, PrPC overexpression using an adenoviral vector increased PrPC and α7nAChR protein expression levels. Overexpression of PrPC upregulated α7nAChR-mediated autophagic flux and activated α7nAChR signaling to prevent PrP(106-126)-mediated neurotoxicity in PrPC knockout Zpl 3-4 cells. These observations support the hypothesis that PrPC regulates α7nAChR signals and that activation of α7nAChR signals protects neuronal cells from prion-mediated neurotoxicity by regulating the autophagy pathway.

This is the first report demonstrating that α7nAChR signaling is regulated by PrPC expression and that upregulation α7nAChR signaling may be related with autophagy signals that protect against PrP(106-126)-mediated neurotoxicity. These results suggest that upregulation of PrPC and inducers of α7nAChR, including nicotine and PNU-282987, may be useful neurotherapeutic strategies for neurodegenerative diseases, including Alzheimer's, Parkinson's, and prion diseases.

## MATERIALS AND METHODS

### Materials

Penicillin-streptomycin solution, trypsin-ethylene diamine tetra acetic acid (EDTA) solution, Neurobasal Medium (NBM), B27 supplement, Glutamax and fetal bovine serum (FBS) were obtained from Life Technologies/Gibco (Carlsbad, CA, USA). Minimum Essential Medium (MEM) and Dulbecco's Modified Eagle's Medium (DMEM) were purchased from GE Healthcare/HyClone (Logan, UT, USA). Methyllycaconitine citrate salt (MLA) and PNU-282987 were purchased from Sigma-Aldrich (St. Louis, MO, USA). The immunoblotting and immunocytochemistry antibodies targeted LC3 and cleaved-caspase-3 (*Cell Signaling* Technology, Danvers, MA, USA), α7nAchR (*Abcam* Inc., Cambridge, MA, USA), p62 (Millipore, Temecula, CA, USA) and β-actin (*Sigma-*Aldrich).

### Primary neuron cell cultures

BALB/c mice brains were isolated from E-15 mice embryos using surgical procedures approved by the Institutional Animal Care and Use Committee of Chonbuk National University. Brain tissues were isolated under sterile conditions, rinsed in HBSS, and minced into small pieces. After dissection, the brain tissues were dissociated in trypsin and plated on poly-D-lysine-coated 24-well plates in MEM, containing 10% FBS. The medium was replaced with NBM supplemented with B27 and Glutamax after 2 hr. All cell cultures were maintained at 37°C in 5% CO_2_.

### Cell culture

The mouse neuronal cell lines ZW 13-2 and Zpl 3-4, which were established from the hippocampus of ICR (*Prnp*^+/+^) and Zürich I *Prnp*^−/−^ mice, respectively, were kindly provided by Professor Yong-Sun Kim (Hallym University, Chuncheon, Kangwon-do, South Korea). The cells were grown in DMEM containing 10% FBS and gentamycin (0.1 mg/ml) in a humidified incubator maintained at 37°C with 5% CO_2_.

### PrP (106-126) treatment

Synthetic PrP (106-126) (sequence, Lys-Thr-Asn-Met-Lys-His-Met-Ala-Gly-Ala-Ala-Ala-Ala-Gly-Ala-Val-Val-Gly-Gly-Leu-Gly) was synthesized by Peptron (Seoul, Korea). The peptide was dissolved at a concentration of 12.5 mM in sterile DMSO and stored at −80°C.

### Annexin V assay

Apoptosis was assessed with the Annexin V assay in detached cells using the Annexin V Assay kit (Santa Cruz Biotechnology, Santa Cruz, CA, USA), according to the manufacturer's protocol. Annexin V was quantified by measuring fluorescence at an excitation wavelength of 488 nm and an emission wavelength of 525/30 nm using a Guava EasyCyte HT (Millipore)

### Terminal deoxynucleotidyl transferase dUTP nick end labeling (TUNEL) assay

The TUNEL analysis was performed to measure the degree of cellular apoptosis using the APO-BrdU™ TUNEL Assay Kit (Invitrogen), following the manufacturer's instructions. Cells were washed with phosphate buffer saline (PBS) and fixed in paraformaldehyde for 15 min. Cells were pre-incubated with 50 μL DNA-labeling solution (10 μL TdT reaction buffer, 0.75 μL TdT enzyme, and 8 μL Br-dUTP) for 1 hr at 37°C and then incubated with 5 μL anti-BrdU-FITC antibody for 30 min at room temperature (20°C). Finally, the cells were mounted with DakoCytomation fluorescent medium and visualized under a fluorescent microscope (Olympus, Tokyo, Japan). The cells were counterstained with propidium iodide to reveal cell nuclei.

### Western blotting

Cells were lysed in lysis buffer (25 mM HEPES; pH 7.4, 100 mM NaCl, 1 mM EDTA, 5 mM MgCl_2_, 0.1 mM DTT, and a protease inhibitor mixture). Proteins were electrophoretically resolved by 10-15% sodium dodecyl sulfate-polyacrylamide gel electrophoresis (SDS-PAGE), and immunoblotting was performed as described previously. Equal amounts of lysate protein were resolved by 10-15% SDS-PAGE and electrophoretically transferred to a nitrocellulose membrane. Immunoreactivity was detected through sequential incubation with horseradish peroxidase-conjugated secondary antibodies and enhanced chemiluminescent reagents. Densitometry of the signal bands was conducted using the Bio-1D densitometer (VilberLourmat, Eberhardzell, Germany). The antibodies used for immunoblotting were α7nAchR, p-62, LC3, PrPC, and Δ-actin. Images were examined using a Fusion-FX7 imaging system (Vilber Lourmat).

### Immunocytochemistry

The cells were cultured on glass cover slips, washed with PBS, and fixed in cold acetone for 90 s at room temperature. They were washed again with PBS, blocked with 5% FBS in Tris buffered saline with Tween, and incubated with anti-mouse-p-62 (2 μg/ml) monoclonal antibody and anti-rabbit-LC3 (2 μg/ml) polyclonal antibody for 48 hr at room temperature. Unbound antibody was removed by an additional PBS wash, and the cells were incubated with Alexa Fluor 488 anti-rabbit FITC (for the anti-LC3 and anti-α7nAchR antibodies) and Alexa Fluor 546 anti-mouse (for the anti-p62 antibody) IgG antibodies (4 μg/ml) for 2 hr at room temperature. Finally, the cells were mounted using the DakoCytomation fluorescent medium (DAKO, Glostrup, Denmark) and visualized under a fluorescence microscope (Olympus).

### RNA interference

The cells were transfected with ATG5 small interfering RNA and α7nAchR small interfering RNA (siRNA; oligoID HSS114104 and oligoID HSS101914; Invitrogen) using Lipofectamine 2000, according to the manufacturer's instructions, respectively. After a 48 h culture, knockdown efficiency was measured at the protein level by immunoblot. The RNAi Negative Control (Invitrogen) was used as a control.

### Adenoviral vectors

Recombinant adenoviruses expressing the full length PRNP gene (Ad-PRNP) were synthesized by Genenmed (Seoul, Korea). A recombinant adenovirus lacking an expression cassette (Ad-empty) was used as a control. The cells were transfected with Ad-PRNP and Ad-LacZ in MEM media without FBS for 24 hr. DMEM with 2% FBS was added after washing with sterile PBS buffer.

### Statistical analysis

All data are expressed as mean ± standard deviation and compared using one-way analysis of variance with GraphPad Prism ver. 5.0 software (GraphPad Software, Inc., La Jolla, CA, USA). A *P* < 0.05 was considered significant.
